# Hepatocellular Carcinoma: Current Therapeutic Algorithm for Localized and Advanced Disease

**DOI:** 10.1155/2022/3817724

**Published:** 2022-12-31

**Authors:** Anju Jose, Maria Grazia Bavetta, Erika Martinelli, Fabrizio Bronte, Emilio Francesco Giunta, Kanjoormana Aryan Manu

**Affiliations:** ^1^Department of Immunology, Amala Cancer Research Centre, Thrissur 680555, Kerala, India; ^2^HepatOncology Section, Internal Medicine Unit, Department of Medicine, Ospedali Riuniti Villa Sofia-V. Cervello, 90100 Palermo, Italy; ^3^Medical Oncology Unit, Department of Precision Medicine, Università Degli Studi Della Campania “Luigi Vanvitelli”, 80131 Naples, Italy; ^4^Gastroenterology Unit, Department of Medicine, Ospedali Riuniti Villa Sofia-V. Cervello, 90100 Palermo, Italy; ^5^Department of Experimental Medicine, Università Degli Studi Della Campania “Luigi Vanvitelli”, 80138 Naples, Italy

## Abstract

Hepatocellular carcinoma (HCC) is the most common primary liver cancer in patients with liver cirrhosis of various etiologies. In recent years, there has been an advance in the knowledge of molecular mechanisms and a better staging definition of patients which has allowed the development of new therapies that have entered the therapeutic workup of these patients. Deep information on molecular drivers of HCC contributed to the development of targeted therapies with remarkable benefits. The novel strategies of targeting immune evasion using immune checkpoint inhibitors and CAR-T and TCR-T therapeutics have also shown promising results. For advanced diseases, the therapeutic algorithm has been recently updated, thanks to the efficacy of combining immunotherapy and antiangiogenic therapy in the first-line setting, and new drugs, both as single-agents or combinations, are currently under investigation.

## 1. Introduction

The primitive liver tumor is represented in the vast majority of cases by hepatocellular carcinoma (HCC) and mainly arises in cirrhotic patients but can develop in a small percentage of cases even in chronic hepatitis. The principal factors responsible for the development of HCC are represented by viral infections of hepatitis B viruses (HBV) and by the hepatitis C virus (HCV) or by metabolic causes such as nonalcoholic steatohepatitis (NASH). In the last years, the treatment of HBV and HCV has significantly reduced the incidence of HCC; however, the risk of developing the tumor persists above all in male and cirrhotic patients. The estimates of the greatest tumor registers report HCC as the sixth cause of death in the world with an estimated world annual impact of around 500,000–1,000,000 cases, with wide variability depending on the geographical area. This determines great attention to patients with chronic liver diseases and an active surveillance for the search for cancer in very early phases in order to guarantee a healing treatment. This review aims to review the molecular mechanisms at the base of the HCC, the staging system currently used, and the therapeutic options.

## 2. Epidemiology in Liver Diseases

Hepatocellular carcinoma in most cases arises from chronic liver disease caused by HBV, HCV, or NASH. The main carcinogenetic factor is represented by HBV. Thanks to the compulsory introduction of HBV vaccination and the use of suppressive antiviral therapies (NUCs) in patients with chronic liver diseases, the incidence has significantly reduced. However, the patients with chronic HBV infection with long-term viral suppression on NUCs are still at risk of developing hepatocellular carcinoma at installments for a year that varies from 0.1% to 1.4% in patients without cirrhosis and from 1.5% to 5.4% in patients with cirrhosis [1]. Another carcinogenic factor is represented by chronic HCV infection. The data show that the annual incidence of HCC in patients with HCV and cirrhosis is approximately 1–4%. In recent years, thanks to the introduction of direct-acting antiviral drugs (DAA), which has allowed the eradication of HCV, the incidence has decreased. However, the question of the recurrence of HCC in those patients who have eradicated the infection still remains open [2]. In recent years, thanks to vaccination programs and therapies for HBV and HCV, there has been an increase in the incidence of HCC in patients with chronic metabolic liver disease, especially in the geographical areas of North America and Europe. In NASH, HCC can develop both in patients with cirrhosis and also in patients without cirrhosis sometimes presenting with an early or intermediate-stage hepatocellular carcinoma, as previously demonstrated [3] and in about 20–30% and it seems that diabetes and obesity are the additional risk factors for the development of this neoplasm [4]. In this patient setting, it seems that some genetic factors and in particular some single nucleotide polymorphism (SNPs) predispose to the development of HCC, for example, the rs738409 variants in patatin-like phospholipase domain-containing 3 (PNPLA3) and rs58542926 variants in transmembrane 6 superfamily member 2 (TM6SF2) (4). Although the incidence of HCC has been reported to be low in autoimmune hepatitis, in a recent systematic review and meta-analysis, the incidence of HCC in chronic autoimmune liver disease was found to have an overall incidence rate of 3.06 per 1000 patient-years (95% CI, 2.22–4.23). Advanced age, male gender, and the presence of cirrhosis at the time of diagnosis are surely the predominant risk factors for HCC. Also, paying attention to autoimmune forms and advocating the importance of regular monitoring of disease severity in AIH [5]. In this modified scenario, alcohol played an important role as a promoter of inflammation and fibrosis favoring the progression to cirrhosis and increasing the risk of HCC [6].

## 3. Molecular Mechanisms and Possible Targets

The molecular pathogenesis of HCC is influenced by a number of genetic and epigenetic alterations. TP53, TERT promoter, CTNNB1, AXIN1, ARID1A, NFE2L2, ARID2, and RPS6KA3 were shown to have the highest mutations and chromosome changes. The overexpressions of EGFR, RAS, FGF, PDGF, VEGF, TGF, mTOR, WTPAP, estrogen receptor, C-MET, CSF-1, and PDL-1 also contribute to HCC progression. Various attempts have been made to therapeutically target these altered proteins and pathways and some of them generated promising results.

### 3.1. Targeting Mutated Genes in HCC

#### 3.1.1. TP53 Mutations

TP53 mutations were frequently seen in poorly differentiated tumors with lot of vascular invasions, cell proliferation, angiogenesis, and epithelial-to-mesenchymal transition [7]. Aflatoxin B1 (AFB1), a mycotoxin, can cause p53 mutations in HCC by inducing G : C to T : A transversions at the third base in codon 249 [8]. Chronic HBV, HCV infections as well as oxyradical illnesses such as hemochromatosis, also can alter TP53 [9]. HBx, a viral protein involved in viral genome transcription, can bind to p53 and inhibit the DNA binding activity of p53 causing hepatocellular carcinogenesis [9, 10]. TP53 mutations, particularly the hot variants R249S and V157F, are linked to a poor prognosis of HCC [11]. When paired with mitotic error-generating mutations such as spindle checkpoint faults and/or Rb deficiency, loss of p53 function can allow aneuploid cells to survive or propagate [12, 13]. High mitotic error-mediated chromosomal instability (CIN) seen in HCC is linked to p53 mutation and hepatitis virus infection [14]. A recent study found an abundance of mutations in TP53, RB1, and SF3B1 in advanced HCC and patients with a poor prognosis, indicating that mutations in genes governing the cell cycle (TP53, RB1) and the spliceosome machinery (SF3B1) play a significant role in cancer progression [15].

#### 3.1.2. TERT Mutation

TERT (telomerase reverse transcriptase) is a rate-limiting catalytic subunit of telomerase that is required for telomere length maintenance and plays a key role in stem cells, aging and cancer. Telomerase depletion and telomere shortening are linked to DNA double-strand breaks, genomic instability, and aging. Overexpression of telomerase lengthens telomeres and facilitates immortalization, which is beneficial to cancer cell survival [16]. TERT expression is typically suppressed in somatic cells, in contrast, 90 percent of cancer cells maintain stable expression of this enzyme [17]. The TERT gene is found on chromosome 5p15 in humans and in HCC, this gene (including the promoter region) has a 60% mutation rate [18]. In HCCs, most of the mutations are either C228T or C250T (reviewed by the authors of reference [19]). The TERT promoter region is 260 base pairs long and contains numerous transcriptional factor binding sites, including MYC and sp1. The TERT promoter mutation is one of the most common changes in HCC [11], which provides several potential binding sites for E-twenty-six/ternary complex factors (ETS/TCF) transcription factors and boost promoter activity [20, 21].

There is no drug available yet to target TERT specifically. A TERT DNA vaccine INO-1400 is tested in a phase 1 trial for solid tumor patients (NCT02960594) [22]. Another vaccine study using a TERT-derived peptide (TERT461) was found to induce TERT-specific immunity in 10/14 (71.4%) patients. 57.1% of patients treated with TERT461 peptide-specific T cells successfully prevented HCC recurrence [23]. Using other telomerase inhibitors, such as the small molecule inhibitors RHPS4 and BRACO-19, as well as PARP inhibitors, also received scientific attention [24]. TERT mutations were shown to be strongly related with CTNNB1 mutations in HCC by certain researchers, implying that the interaction between TERT mutations and dysregulation of WNT-catenin pathways could enhance HCC malignancy [11].

#### 3.1.3. WNT-*β*-Catenin Pathway

WNT signaling is an important regulator of the developmental process, tissue homoeostasis, and stem cell proliferation in metazoans. During canonical signaling (*β*-catenin dependent), WNT ligand bound to a Frizzled receptor and its coreceptor (LDL-receptor-related protein (LRP) 5/6) results in a receptor complex formation which stabilizes *β*-catenin and boosts transcription of cell-growth genes [25]. WNT absence causes the inactivation of *β*-catenin by the formation of a destruction complex that comprises of glycogen synthase kinase 3 (GSK3), adenomatosis polyposis (APC), AXIN, and casein kinase 1 alpha which phosphorylates and degrade *β*-catenin by ubiquitin-mediated proteasomal degradation [26–28]. Stimulation of beta-catenin and WNT signaling also may lead to genomic instability, particularly when accompanied by an increase in DNA damage or mismatch repair errors, which are common in the development of HCC [16]. Mutations in the catenin beta 1 (CTNNB1) are observed in 20–30% of cases of HCCs which include conventional mutations in codons 33, 37, 41, and 45 and nonconventional missense mutations in codons 32, 34, and 35 (reviewed by the authors of reference [29]). One study revealed a somatic mutation of CTNNB1 at exon 3 that enhances protein stability and expression of *β*-catenin significantly by inhibiting phosphorylation and ubiquitination [30].

Among WNT pathway genes, AXIN1 is the second most frequently mutated with a frequency of 9.6% in HCC [11]. AXIN1 acts as a negative regulator of WNT/-catenin signaling by encouraging phosphorylation and degradation of *β*-catenin. The double mutations CTNNB1 and AXIN 1 show increased DNA binding of TCF associated with *β*-catenin in the nuclei. Axin is reported to be an effective molecule for suppressing the growth of hepatocellular and colorectal cancers [31]. The small chemical inhibitor XAV939 has been proposed as a novel approach to target the WNT pathway by stabilizing AXIN by decreasing the poly-ADP-ribosylating enzymes tankyrase 1 and tankyrase 2 [21].

#### 3.1.4. ARID (AT-Rich Interactive Domain-Containing Protein)

ARID1A (AT-rich interactive domain-containing protein 1, also known as BAF250a) is a member of the switch (SWI)/sucrose nonfermenting (SNF) family and are considered to regulate the transcription of specific genes by modifying the chromatin structure [16]. The expression of the SWI/SNF complex in normal liver tissues is high and acts as a tumor suppressor gene, preventing cell proliferation [11]. According to Samartzis et al, the PI3K/AKT pathway activation is also a critical mechanism in ARID1A-mutated malignancies, and ARID1A-deficient tumors are preclinically sensitive to the PI3K/Akt/mTor pathway inhibitors [32].

Another most frequently mutated gene in SWI/SNF complex is AT-rich interactive domain 2 (ARID2). ARID2 inhibit cellular proliferation and growth of HCC cells and mutations cause inactivation. There are 5–10% of HCCs having mutations in ARID2 [33]. The mutations in ARID 2 include nonsense mutations, frame-shifting indels, and splice site mutations [34]. ARID2 is involved in chromatin remodelling-mediated transcriptional activation and repression of certain genes. Alcohol intake-related HCCs were more likely to have ARID1A mutations, while HCV-related HCCs were more likely to have ARID2. (Ding et al. [11].

#### 3.1.5. KEAP1 and NFE2L2

Mutations in Kelch-likeECH-associated protein 1 gene (KEAP1)/nuclear factor, erythroid 2-like 2 genes (NFE2L2) are found in advanced HCC [35]. NFE2L2 is a mediator of the transcription of genes encoding detoxifying enzymes, and antioxidant and multidrug resistance proteins and activation of NFEL2L can cause chemotherapy and radiation resistance in cancer cells [36–38]. KEAP1 induces proteasomal degradation of NFE2L2. KEAP1 mutation results in constitutive activation of NFE2L2 and overexpression of its target genes, which results in cancer cell survival [25]. It is reported that NFE2L2 abnormalities in the oxidative stress pathway enhance hepatocarcinogenesis by cooperating with the WNT-*β*-catenin pathway [11].

### 3.2. Targeting Differentially Expressed Genes/Proteins in HCC

#### 3.2.1. EGFR/RAS

Even though RAS mutation is associated with only 7% of HCC, the RAS-associated pathways appear to be overactive in HCC mostly due to enhanced EGFR expression and activity. EGFR/RAS signalling-induced RAF-MEK-ERK and PI3K/Akt/mTOR kinase cascades contribute to the development and maintenance of HCC. Multikinase inhibitors which can inhibit various kinases in the EGFR or RAS downstream kinase cascade gained attention globally. Even though multikinase inhibitors such as sorafenib has shown remarkable progress in HCC treatments, the overall outcome was not satisfactory.

The development of sorafenib resistance was another hurdle in the HCC treatment strategy but the use of combinations has shown some promising results. HCC patients treated with the MEK1/2 inhibitor refametinib + sorafenib showed a greater clinical response than those treated with refametinib alone, especially those with RAS mutations [39]. The anticancer activity of sorafenib could be enhanced by combining it with rapamycin or aspirin to inhibit the mTOR signaling pathway. THOR (testis-associated highly conserved oncogenic long noncoding RNA), a novel LncRNA, was recently identified as an activator of the downstream WNT-catenin signaling pathway, implying that HCC patients with low THOR expression may benefit more from sorafenib treatment [40, 41]. Common protein-level activation of the RAS/RAF/MAP-kinase pathway as well as rare somatic mutations such as RPS6KA3 mutations (2–9 percent) and KRAS mutations, have been reported in human HCC (1 percent) [42, 43]. MEK inhibitors such as trametinib and refametinib could be used to target this pathway [15].

Overexpression of choline kinase has been shown to increase functional interaction between EGFR and mTORC2 for Akt activation, promoting HCC metastasis and treatment resistance to EGFR inhibitors gefitinib and erlotinib [44].

#### 3.2.2. mTOR

Tumors with poor differentiation, vascular invasion, and high stages, as well as those with poor prognosis, show more changes in the mTOR pathway [11, 45]. HCC associated with CK19 expression has shown elevation of numerous cancer stem cell/progenitor genes and PI3K/AKT pathway activation,TSC1/TSC2 mutations and phospho-S6 overexpression [7]. The PI3K/AKT pathway inhibitors can be a promising strategy for patients with this specific variation [7].

#### 3.2.3. Estrogen Receptors

Estrogen receptors also have a role in regulating HCC tumorigenesis and progression, although the role and mechanism of GPER in the development and advancement of HCC are unknown. GPER was found to be downregulated in HCC tissues compared to nontumor equivalents, and GPER-specific agonist G1-triggered GPER/EGFR/ERK signalling was found to be important in reducing HCC tumor viability *in vitro* and *in vivo* [46]. GPER/ERK signalling is tightly linked to GPER-positive HCC tissue, and patients who had a high GPER and p-ERK expression at the same time had better clinical outcomes [46].

#### 3.2.4. FGF

The fibroblast growth factor family has 22 ligands and four receptors which are involved in the regulation of cell survival, proliferation, migration, differentiation, embryonic development, organogenesis, tissue repair, regeneration, and metabolism by regulating mostly RAS/STAT3/WNT signalling. In mature liver cells, FGF19 is mutated in approximately 4 to 6 percent of patients with HCC and affects the PI3K-AKT-mTOR pathway. FGF19 is also a biomarker for HCC.

CCND1 (coding cyclin D1) is located on chromosome 11q13 [11]. 11q13.3 amplification also could be a biomarker for patients who are likely to respond to anti-FGF/FGFR drugs [47]. An anti-FGF19 antibody as well as RNAi-mediated suppression of FGF19 or CCND1 inhibited clonal development and tumorigenicity of HCC cells carrying the 11q13.3 amplicon. Blocking the interaction between FGF19 and FGFR4 through the use of an anti-FGF19 monoclonal antibody effectively prevented HCC in transgenic mice [48]. In a limited subset of patients with HCC, lenvatinib, an oral tyrosine kinase inhibitor that targets FGFR1–4, VEGFR1–3, KIT RET, and PDGFR-beta has generated partial responses. But the FGFR inhibitor brivanib did not enhance survival in unselected individuals with HCC [24].

#### 3.2.5. VEGF

Vascular endothelial growth factor A (VEGFA), a key player in promoting the proliferation and migration of endothelial cells and improving vascular permeability has shown higher mRNA and protein levels in HCC tumor cells than those in paracarcinoma tissue [49]. In HCC patients, high levels of VEGF are related to poor overall survival rate (OS) and lower progression-free survival (PFS). VEGFR inhibitors cabozantinib and ramucirumab show an antitumor activity in HCC through interdicting VEGFR-2 [11]. Angiopoietin-2 is a mediator of vascular remodelling and it can induce neoangiogenesis and endothelial sprouting in conjunction with VEGFA [50]. A combination of both angiopoietin, another mediator of vascular remodelling and neoangiogenesis, and VEGFA inhibitors have shown exceptional efficacy in HCC treatment [7, 51].

#### 3.2.6. PDGF

Platelet-derived growth factor (PDGF) and PDGFR, another angiogenesis modulator have an intimate relationship with the development and progression of HCCs. There are four PDGF ligands (PDGF-A,-B,-C,-D) that bind to two different receptors, PDGFR *α* and PDGFR *β*. In HCC tissues, PDGFR *α* is upregulated when compared with adjacent liver tissues [52]. PDGF-A shows a consistent increase with PDGFR*α* expression and it is found that those with high expression of PDGFR*α* have a significantly lower overall survival and worse disease-free survival (DFS) with a *p* value of 0.005 and 0.025, respectively [52]. Moreover, PDGF-C, another ligand binding with a high affinity to PDGFR*α*, plays a key role in liver fibrosis by stimulating mesenchymal cell types, and also stellate cells are included. And there is a positive correlation between PDGF-C expression level and HCC staging and PDGF-C also activates intracellular signalling pathways involving PKB/Akt [53]. All of these studies indicate that the PDGF/PDGFR system may be a potential therapeutic target [11].

#### 3.2.7. TGF Pathway

TGF-*β* family of cytokines has been composed of 33 members in mammals (25). They have been subdivided into the TGF-*β* subfamily which include, TGF-*β*, activin nodal and lefty and BMP subfamily which include bone morphogenetic proteins (BMPs) and growth and differentiation factors (GDFs). TGF pathway comprises of a very complex network which includes both SMAD-dependent and SMAD-independent pathways [54]. The transforming growth factor (TGF) has a dual function, which can inhibit cell proliferation and induce apoptosis in primary tumors while promoting tumor progression and metastasis in the advanced stages by enhancing cell motility, EMT, invasiveness, and stemness [55–57]. TGF beta pathway regulation in liver cancer has been beautifully reviewed by Tu et al. [54]. The HBV/HCV infections can contribute to rewiring the TGF beta pathway helping tumorigenesis [58–62].

TGF signalling is also a critical modulator of immune cell proliferation, differentiation, development, and survival, suppressing CD8^+^ T cells, NK cells, and DCs while increasing CD4^+^CD25^+^ Tregs by boosting M2-type macrophage differentiation, resulting in immunosuppression in HCC. TGF generates a favorable condition for tumor development and metastasis by altering the microenvironment [57].

#### 3.2.8. WTAP

The gene Wilms' tumor 1-associating protein (WTAP) is found on chromosome 6q25-27. WTAP is a nuclear protein that regulates the cell cycle, proliferation, apoptosis, RNA splicing, and N6-methyladenosine RNA modification (m6A) in numerous physiological and pathological processes [63]. WTAP promotes the progression of HCC in an m6A-dependent way and the miR-139-5p/WTAP axis has a role in HCC advancement by regulating the EMT. miR-139-5p/WTAP axis has been shown to be a diagnostic and therapeutic target against HCC [64].

#### 3.2.9. C-MET

C-MET, a receptor tyrosine kinase which can bind with hepatocyte growth factor has been shown to have abnormalities in almost 50% of HCC cases (80) [65] and is involved in HCC progression [66]. Selective c-MET inhibitors have been reported to be promising (NCT02795429, NCT02082210) over nonselective inhibitors (NCT01988493, NCT02115373; NCT01737827, NCT01964235) (reviewed by [57]. A c-MET inhibitor tivantinib reported to have an effect on HCC and Child-Pugh A or B cirrhosis [67]. The safety profile was manageable with prompt therapies. The best response was stable disease in 56% of patients with a median time to progression of 3.3 months.

### 3.3. Targeting Immune System against HCC

Immune checkpoints can be activated by the tumor cells which results in antitumor immunity. Inhibition of the immune checkpoints recently received lots of attention which led to the development of several immune checkpoint inhibitors. Many such immune checkpoint inhibitors have been approved for the treatment of various cancers. Antibodies against cytotoxic T-lymphocyte-associated protein 4 (CTLA4) and antibodies that inhibit programmed cell death 1 (LD1) or PDL-1 are among them.

#### 3.3.1. CTLA4

CTLA4 is an immunoglobulin family member which is expressed in activated T cells. Unlike the other T cell costimulatory molecule CD28, CTLA can transmit inhibitory signals to T cells [68]. CTLA4 has been constitutively expressed in Treg (Regulatory T) cells [69]. Anti-CTLA4 antibody tremelimumab was the first immune checkpoint inhibitor tested. In phase 2 [70]), HCC patients with HCV infection and ineligible for surgery showed a partial response with an overall survival of 8.2 months. The combination with conventional therapies also showed a partial response of 26% [71, 72].

#### 3.3.2. PD-1 and PD-L1 Inhibitors

Programmed death ligand 1 (PDL-1) is a ligand which can bind with PD-1 receptors of cells in the adaptive immune system such as T and B cells and activate programmed cell death of them. The expression or overexpression of PDL-1 makes the tumor cells less susceptible to T or B cell targeting and they will be able to escape from the immune reaction. Some of the meta-analysis studies show a correlation between PDL-1 expression and poor prognosis while some are inconclusive. Some studies [73–78] have shown that the expression of PDL-1 is correlated with a poor prognosis after hepatectomy, whereas other studies have reported nonhomogeneous results [79–82]. The combination of anti-CTLA4 and anti-PD-1/PDL-1 are also being tested and results are awaiting [83]). Inhibitors of other check point regulators such as BTLA, TI3, and LAG3 are also in trials against cancers including HCC (NCT03005782, NCT03489369, NCT01968109, NCT03250832, NCT03489343, NCT02817633, and NCT03099109) [72].

#### 3.3.3. CAR-T Therapy

Chimeric antigen receptor-T cell therapy is a novel strategy in adoptive cell transfer (ACT) which is one of the promising fields in immunotherapy. During CAR-T therapy, the T cells are being genetically modified to recognize specific tumor associated antigen. Gao et al. demonstrated CAR-T cells targeting GPC3, a heparan sulfate proteoglycan which is not expressed in normal liver but overexpressed in HCC, effectively eliminating the growth of HCC in *in vitro and in vivo* [84]. A different study by Jang et al. [85], showed that the expression of PDL-1 on tumor cells contributes to the variation in therapy efficiency of GPC3 CAR-T cells. Another attempt to make CAR-T cells which target AFP (alpha-fetoprotein) 158–166 peptide-MHC complex in HCC is on progress and already finished a phase 1 trial [86]). Another study found successful lyse of HCC by using CAR-T cells targeting NKG2DL [87]. CD147 [88] and CD133 [89–91] are also found to be effective CAR-T targets in HCCs. Identification of more and tumor-specific targets for CAR-T may improve the therapeutic potential and patient recovery in the recent future.

#### 3.3.4. TCR Engineered T (TCR-T) Cells

TCR-T cells are coined for the T cells modified with exogenous TCRs in order to specifically target the tumor antigen peptide-MHC complex (92). One of the advantages of TCR-Tover CAR-T cells are that they can recognize tumor antigens that are on the tumor surface or intracellular. Spear et al. in 2016 [92] genetically developed HCV-specific TCR-T cells which could induce regression of established HCV + HCC *in vivo*. HBV-specific TCR-T cells [93] were found to recognize HBV-associated HCC cells. Additionally, they were found to be preventing the recurrence of HCC after liver transplantation [94] NCT02686372, [95].

#### 3.3.5. CSF-1/CSF-1R

Most of the recent developments in tumor immunology are based on the idea that the innate immune system contributes to the tumor growth and metastatic spread of cancer. Colony-stimulatingfactor-1 is one such innate immunity-regulating cytokine which is secreted by tumor cells and helps to recruit macrophages. CSF-1-activated tumor-associated macrophages can contribute to tumor growth and metastasis. PLX3387, a CSF-1R inhibitor has been shown to have an antitumor effect in both xenograft and allograft HCC tumors. There are several TKIs including such as pexidartinib (NCT02452424), chiauranib (NCT03245190), TPX-0022 (NCT03993873), and BLZ945 (NCT02829723) which are known to have antitumor effects against solid tumors including HCCs [57, 96–99].

## 4. Hepatocellular Carcinoma: The Staging Dilemma and the Latest BCLC Update

**Figure 1:**
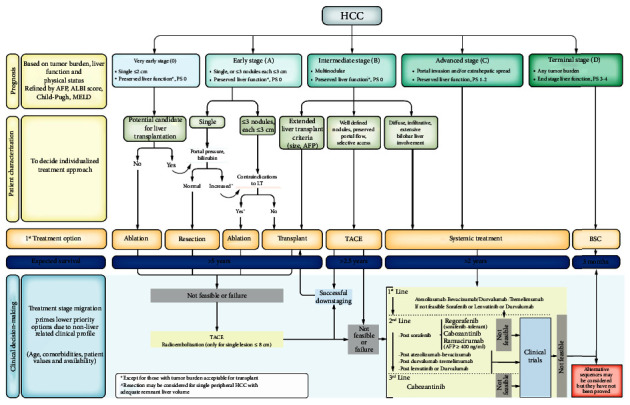
BCLC update 2022 staging system [103].

Hepatocellular carcinoma (HCC), in the vast majority of cases, arises from a chronic liver disease (CLD) of various etiology, the most frequent forms of which are those to viral etiology (HCV and HBV) and in recent years metabolic ones (NASH). The choice of tumor treatment depends not only on the stage of the tumor but also on the degree of impairment of the underlying CLD, which in itself can influence the choices for tumor therapy. In the last 40 years, we have tried to create a score, albeit with different problems, which could unify the two parameters and give a staging system that guarantees the therapeutic choice and predicts prognosis and survival. The first was the TNM staging system it evaluates only tumor parameters and does not include liver function and so does not provide a precise prognosis for patients in different risk groups [100]. In 1984, it was the turn of the Okuda Score which included both tumour and liver function factors but is no longer useful as most of the HCCs are detectedearly [101]). In 1998 a group of Italian researchers proposed the Cancer Liver Italian Program score (CLIP) which used four variables: the Child-Pugh, the morphology of the neoplastic mass, the serum levels of alpha-fetoprotein, and the possible presence of thrombosis of the vein. This system was also gradually abandoned as its main limitation is the impossibility of identifying very early-stage patients who could benefit from curative treatments (liver resection and percutaneous treatments, RFTA and TACE). Another limitation is the difficulty of stratification, as only three stages are contemplated [102]. In 1999 two staging systems have been proposed: the first by the Barcelona group that proposed Barcelona Clinic Liver Cancer (BCLC) classification then revised in 2003 and later in 2022 after the introduction of systemic treatments. This staging system allows patients to be stratified into 5 staging groups on the basis of dependent variables related to cancer, CLD, and performance status. Therefore, this staging system allows us to decide the therapeutic strategy to be adopted and to define prognosis and survival [103]. The second by Groupe d'Etude et de Traitement du Carcinome Hepatocellulaire (GRETCH). This staging system stratifies patients into three groups using five parameters: the Karnofsky performance index, the ultrasound documented portal thrombosis, the bilirubin value, the alkaline phosphatase, and alpha-fetoprotein values being very easy to determine but does not guarantee any prognostic value more than other staging systems [104]. In 2002, Chinese University Prognostic Index (CUPI) was proposed. This system considered six variables including TNM, symptoms, ascites, serum AFP, bilirubin, and alkaline phosphatase allowing stratification into three groups. CUPI was considered superior to TNM in predicting patient survival but it did not guarantee the staging of CLD [105]. In 2003, Japanese experts proposed the Japanese Integrated Staging Score (JIS) which involves the use of six stadiative classes by integrating the old TNM classification with the Child-Pugh score. This staging system has only been used in Japan and has never had any validation in countries such as America and Europe [106]. In 2005, the Tokyo score was proposed, which was validated only on 403 Asian patients and exploits four factors: serum albumin, bilirubin, and the size and number of tumors. Just as the JIS score has the advantage of being easily calculated but does not add an advantage in prognostic terms [107]. In 2010, the Taipei Integrated Scoring system (TIS) was immediately abandoned because it was a score that was mainly based on total tumor volume (TTV) not evaluating parameters of the underlying disease and was conducted retrospectively and not validated in other populations [108]. As a model to estimate survival in ambulatory HCC patients (MESIAH), the MESIAH score provided a better prognostic stratification than other staging system in treated HCC patients. It was not helpful in predicting the natural course of HCC [109]. Hong Kong Liver Cancer (HKLC) is a score developed in 2014 and is based on some criteria, performance status, Child-Pugh score, tumor status, vascular invasion, and metastasis and were weighted with a relative coefficient. However, this score has only been validated on patients with chronic HBV-HCC infection and is able to prognostically stratify patients in the intermediate and advanced stages. Like many of the scores, it does not have validation in Europe and North America [110]. Italian Liver Cancer ITA.LI.CA is a score created in 2016 based on tumor size, liver function, and other patient-related variables and a difference between the other scores that favors a better definition of the intermediate-stages of HCC [111]. A model to estimate survival for hepatocellular carcinoma MESH, introduced in 2016, is a score based on the Milan criteria, the presence and type of vascular invasion, C-P score, performance status, and laboratory parameters (AFP and alkaline phosphatase). One of the flaws of this system is the lack of therapeutic options but unlike many other scores, it has external validation in Europe and America [112]. CNLC, which was introduced in 2017 and subsequently revised in 2019, is an analogue of BCLC and uses the same prognostic factors as the BCLC (patient's general health status, tumor burden, and liver function) with some options provided for both early and even for the advanced stages [113]. From this brief overview of HCC staging systems, it is possible to understand how difficult it is to create a score that is able to optimize patient staging and guarantee prognosis and survival. Currently, BCLC is the staging system adopted for prognosis, survival, and therapeutic choice and it is adopted by the most important scientific societies of chronic liver diseases, American (AASLD) [114] and European (EASL) [115]. It has been identified as the best staging system because it is able to predict the prognosis and mortality of patients by presenting the three best predictors of prognosis which are represented by the extent of the tumor, the severity of the disease, and the state of health of the patient [116]). In 2022, the BCLC was updated. Currently, BCLC, using the following parameters relating to the tumor (size, number, and vascular invasion), liver function (Child-Pugh) and performance status, has five stages (Figure 1):

- Very early stage (0) (single ≤2 cm, preserved liver function, PS 0)
- Early stage (A) (single, or ≤3 nodules each ≤3 cm, preserved liver function, PS 0)
- Intermediate stage (B) (multinodular, preserved liver function, PS 0)
- Advanced stage (C) (portal invasion and/or extrahepatic spread preserved liver function, PS 1-2)
- Terminal stage (D) (Any tumor burden end stage liver function, PS 3-4)

In the BCLC, some new concepts were introduced from both a prognostic, diagnostic, and a therapeutic point of view. From the prognostic point of view, the new staging has introduced alpha-fetoprotein (AFP) values as a relevant prognostic factor in it from tumor size. The albumin-bilirubin score was also introduced for the indication of liver function in patients with compensated liver cirrhosis. From the diagnostic point of view, the first novelty of the 2022 revision of the BCLC provides for the introduction of an expert clinical component capable of personalizing the choice based on various variables such as the characteristics of the patient, the tumor, local skills, and the availability of the procedure; The second novelty is represented by the treatment stage migration (TSM). TSM occurs when, due to the patient's characteristics, due to the failure of a treatment or the lack of feasibility of a treatment, the therapeutic option moves towards more advanced therapeutic options, modifying prognosis, and survival. From a therapeutic point of view, the novelties are represented by an extension of the indication for transplantation (LT). In fact, in the previous versions of BCLC the LT was indicated only in multifocal patients, and in the new version, the LT is indicated not only in multifocal patients but also in those with small tumors, in some subgroups of stage B and in those who have had a success of downstaging from locoregional therapies (TACE and TARE); a new indication of transarterial radioembolization (TARE) also finds its place in the new BCLC staging. In fact, TARE is indicated in patients with an early stage (BCLC 0/A) with a tumor smaller than 8 cm and who have no indications for other treatments. One of the most important novelties is represented by the subdivision of stage B into three groups stratified according to the spread of the tumor and liver function. Stage B1 provides for indication to the LT also with an extension of the Milan criteria. Stage B2, patients in whom LT is not indicated but with well-defined nodules and a preserved vascular system may indicate locoregional treatment such as TACE. Stage B3, which includes patients with infiltrating and bilobar cancer, indicates systemic treatment. The last important novelty is represented by systemic therapies. In fact, in this new revision, systemic therapies find their place in the algorithm within patients in stage B3. In these, atezolizumab/bevacizumab or durvalumab/tremelimumab (sorafenib, lenvatinib or durvalumab if unavailable) indicate as first-line treatment; second-line treatment with regorafenib or cabozantinib, ramucirumab (after treatment with sorafenib); a third line is represented by cabozantinib [103].

This new update, although it has resulted in an increase in the complexity of the BCLC staging and therapeutic algorithm, has resulted in a better allocation of patients and therapeutic choices, greater dynamism in the treatment of patients, and above all a greater definition of the prognosis and survival of patients. However, future validation studies will be needed to decide whether the BCLC and its 2022 update are really applicable in clinical practice.

## 5. Currently Approved Therapies for BCLC Stage C HCC Patients and Future Perspectives

In the latest years, new drugs have been incorporated into the therapeutic scenario of BCLC stage C HCC. The current algorithm provided by the NCCN guidelines suggests the use of atezolizumab plus bevacizumab as the preferred regimen for the first-line systemic therapy [117].

The association of atezolizumab (anti-PD-L1 monoclonal antibody) and bevacizumab (anti-VEGF monoclonal antibody) has been approved based on the results of the phase III IMbrave150 trial [118], in which the doublet performed better than sorafenib, that was the standard therapy. It must be noted that the association was superior to sorafenib in all the efficacy endpoints (overall survival, OS: 12-months rate of 67.2 vs 54.6%; progression-free survival, PFS: 6.8 vs 4.3 months; objective response rate, ORR: 27.3 vs 11.9%; duration of response, DoR: longer than 6 months in 87.6 and 59.1% of patients, respectively), also delaying the deterioration of patient-reported quality of life. In terms of adverse events, hypertension was the most frequent one in the doublet arm.

Sorafenib and lenvatinib represent two other approved first-line options for BCLC stage C HCC patients [117]. It must be said that, in the era of combination therapies, the use of single-agent tyrosine kinase inhibitors (TKIs) should be restricted to patients with contraindications to immunotherapy (i.e., active autoimmune diseases, patients requiring steroid therapy at high dosages) or in the countries where the doublet is not (yet) available. Sorafenib, an oral antiangiogenic TKI, has been the standard first-line therapy for many years, based on the results from two placebo-controlled clinical trials [119, 120] with a gain in the survival rate of 30%. Lenvatinib, which is also an oral antiangiogenic TKI, demonstrated noninferiority as compared to sorafenib, with a different safety profile, as shown by the REFLECT trial results [121]. Of note, all the abovementioned therapies are recommended for Child-Pugh class A patients only.

Subsequent therapies have been studied in patients progressing to sorafenib. Among antiangiogenic drugs, three molecules have been approved: regorafenib, cabozantinib and ramucirumab; on the other hand, among immunotherapeutic drugs, pembrolizumab (anti-PD-1 antibody) and the combination of nivolumab plus ipilimumab (anti-PD-1 and anti-CTLA-4 antibodies, respectively) received FDA approval in this setting. Regorafenib and cabozantinib are two oral antiangiogenic TKIs which demonstrated a survival improvement over placebo in sorafenib pretreated HCC patients, as reported in the RESORCE and CELESTIAL trials, respectively [122, 123]. Ramucirumab is an anti-VEGFR antibody which demonstrated to prolong survival only in a selected population of HCC patients, that is those with *α*-fetoprotein concentrations of 400 ng/mL or greater [124]. A mention should be also made to the use of chemotherapy, specifically metronomic capecitabine, in patients who failed sorafenib in the first-line setting; capecitabine, which is the oral prodrug of fluorouracil, showed a good safety profile and antitumor activity in a retrospective analysis [125]. Compared to the placebo, pembrolizumab improved survival but without reaching statistical significance per specified criteria in the KEYNOTE-240 trial [126], however, recently, the results from the KEYNOTE-394 trial in Asiatic HCC patients confirmed the OS benefit [127]. Nivolumab, which failed to demonstrate superiority over sorafenib in the first-line setting [128], has shown clinical activity and manageable safety when combined with ipilimumab in pretreated patients [129].

Nowadays, therapeutic algorithms for treatment-naïve BCLC stage C HCC patients should start from the doublet of atezolizumab plus bevacizumab as the preferred first-line approach, but it must be noted that the second-line approved drugs are approved at the time of progression to sorafenib. In 2020, the ASCO guidelines released a therapeutic algorithm in which, after progression with the doublet, a second-line with TKI could be offered [130]. The data from a multinational multicentre retrospective study of HCC patients treated with sorafenib and lenvatinib after the doublet showed a remarkable median OS of 14 months, without significant difference based on the administered drug [131]. The use of TKI after the doublet is supported by several hypotheses: a higher expression of molecular targets (i.e., FGFR4) and a modulation of the tumor microenvironment [132]. Concerning future perspectives, ongoing clinical trials are evaluating the role of new drugs, or new drug combinations, in BCLC stage C HCC patients.

New oral antiangiogenic TKIs have been tested in HCC patients, but to date, most of them have not proven to be superior to already approved therapies. Anlotinib, which displays a pharmacological activity similar to sorafenib, showed a promising activity in the first-line setting in a phase II trial [133]. Apatinib, which has a more selective activity against VEGFR-2 than sorafenib [134], is also a candidate for first-line therapy in HCC patients due to its activity and safety profile [135]. Donafenib is an oral derivative of sorafenib, which is to-date the only new molecule which has performed better than sorafenib in terms of survival; in fact, a phase II-III trial in an Asiatic population has shown a gain in OS rate compared to sorafenib, with fewer grade 3 adverse events [136]; however, donafenib seems to be not cost-effective as compared to sorafenib [137].

It is plausible that upfront TKIs, when administered as a single-agent, are not sufficient to guarantee a meaningful survival improvement, if compared to atezolizumab plus bevacizumab regimen; on the other hand, the only approved association in the first-line setting consists of two intravenous molecules, bevacizumab and a single-target agent. This is the reason why, in the latest years, several combinations of TKI and immunotherapy are being tested in treatment-naïve HCC patients. In the phase I VEGF liver100 trial, 22 treatment-naïve Japanese patients received avelumab (anti-PD-L1 antibody) plus axitinib (oral antiangiogenic TKI) [138]; the combination showed a manageable safety profile, being hypertension and hand-foot syndrome as the most frequent grade 3 AEs, with antitumor activity. In the phase Ib/II AK105-203 trial, the association of penpulimab (anti-PD-1 antibody) and anlotinib was tested in 31 Chinese patients, with a low rate of G3-4 AEs and a remarkable PFS of 8.8 months [139]. The phase II KEEP-Go4 trial evaluated the safety and the efficacy of the combination of anlotinib plus sintilimab (anti-PD-1 antibody) in 20 HCC patients, with a good safety profile (no G4-5 AEs) and potential activity [140]; another phase II trial, the RESCUE trial, evaluated the combination of apatinib plus camrelizumab (anti-PD-1 antibody) in both first and second-line settings, with similar results [140]. In the phase III COSMIC-312 trial, 837 treatment-naïve patients were randomly assigned to the combination of atezolizumab and cabozantinib, cabozantinib as a single-agent or with sorafenib [141]. Despite a clear benefit in PFS of the combination over sorafenib at the first interim analysis, the median OS did not differ between these two arms; however, the long OS recorded in the sorafenib arm suggests the fundamental role of subsequent therapies, once again highlighting the importance of therapeutic algorithms in HCC patients. The most frequent grade 34 AEs were aminotransferase increase, hypertension, and hand-foot syndrome. The phase III LEAP002 trial compared lenvatinib to the association of lenvatinib plus pembrolizumab, waiting for the publication of the first interim analysis, and the sponsor announced that the combination did not meet its coprimary endpoints of PFS and OS [142].

Another strategy that is currently under investigation is to combine two immunotherapeutic agents with different molecular targets, in order to synergistically potentiate the immune system against tumor cells [143]. The ongoing phase III checkmate 9DW trial (NCT04039607) is evaluating the efficacy of nivolumab plus ipilimumab compared to the single-agent TKI (sorafenib or lenvatinib). In case this trial is positive, the question would be if it is better to start with an immuno-immuno combination rather than an immuno-TKI (or immuno-anti-VEGF) combination? and the answer should rely on biomarkers studies, that could better select patients in the next future (Table 1).

It should be noticed that patients enrolled in clinical trials belonged mostly to Child-Pugh class A, being often class B as an exclusion criterion; therefore, patients with Child-Pugh class B have limited therapeutic options. Some studies have already addressed this issue. A retrospective analysis has evaluated the efficacy and safety of nivolumab compared to sorafenib as the first-line therapy for Child-Pugh B HCC patients, highlighting an improvement in survival [146]. Moreover, a retrospective analysis of the aforementioned CELESTIAL trial has shown the safety and efficacy of cabozantinib compared to placebo and also in enrolled patients with Child-Pugh class B score [147]. In conclusion, further prospective studies are warranted in Child-Pugh B patients, to evaluate the best therapeutic algorithm in this population.

## 6. Conclusion

For the last one decade, we are witnessing tremendous growth in the identification of new targets and the blossoming of new strategies for hepatocellular carcinoma treatment which led to the development of many drugs or drug combinations and many more are in the pipeline. In the latest years, progresses in the management of HCC have certainly improved patients' survival rate. However, optimal therapeutic sequencing is far to be defined; the advancement of knowledge in HCC biology will undoubtedly guide further researches in this field, possibly identifying new molecules and drug combinations to be able to overcome pharmacological resistance, which is ultimately the cause of therapeutic failure.

## Figures and Tables

**Table 1:** Ongoing clinical trials in HCC patients.

Drug (s)	Phase	Reference
*New oral antiangiogenic TKIs*		
Anlotinib	II	Sun et al. [133]
Apatinib	II	Hou et al. [135]
Donafenib	II-III	Qin et al. [136]

*Combination of ICIs and antiangiogenic TKIs*		
Avelumab + axitinib	I	Kudo et al. [138]
Penpulimab + anlotinib	Ib-II	Han et al. [139]
Sintilimab + anlotinib	II	Chen et al. [140]
Camrelizumab + apatinib	II	Xu et al. [144]
Atezolizumab + cabozantinib	III	Kelley et al. [141]
Pembrolizumab + lenvatinib	III	Liovet et al. [145]

ICIs, immune checkpoint inhibitors; TKIs, tyrosine kinase inhibitors.

## Data Availability

The data used to support the findings of this study are available from the corresponding author upon request.
